# IL-6 *trans*-Signaling Regulates Neutrophilic Inflammation in Alcohol-Associated Hepatitis

**DOI:** 10.1016/j.ajpath.2025.05.023

**Published:** 2025-06-23

**Authors:** Gaurav Sarode, Ming-Fo Hsu, Fawaz G. Haj, Sergio Barace, Josepmaria Argemi, Mengfei Liu, Prisha Pandita, Sheng Cao, Vijay H. Shah, Dorina Gui, Ramon Bataller, Vikrant Rachakonda

**Affiliations:** ∗ Division of Gastroenterology and Hepatology, University of California–Davis School of Medicine, Sacramento, California; † Department of Nutrition, University of California–Davis, Sacramento, California; ‡ Hepatology Program, Liver Unit, Instituto de Investigación de Navarra (IdisNA), Clínica Universidad de Navarra and Centro de Investigación Médica Aplicada (CIMA), Pamplona, Spain; § Digestive Diseases, Department of Internal Medicine, Yale University School of Medicine, New Haven, Connecticut; ¶ Division of Cardiology, University of California–Davis School of Medicine, Sacramento, California; ‖ Division of Gastroenterology and Hepatology, Mayo Clinic, Rochester, Minnesota; ∗∗ Department of Pathology, University of California–Davis School of Medicine, Sacramento, California; †† Liver Unit, Hospital Clinic, Institut d'Investigacions Biomediques August Pi i Sunyer (IDIBAPS), Barcelona, Spain; ‡‡ Centro de investigación Biomédica en Red en Enfermedades Hepáticas y Digestivas (CIBERehd), Madrid, Spain

## Abstract

Alcohol-associated hepatitis (AH) is a form of acute-on-chronic liver failure characterized by intrahepatic neutrophilic inflammation. In hepatocytes, IL-6 signals through either membrane-bound (classical signaling) or soluble (*trans*-signaling; TS) IL-6 receptors (IL-6Rs) to regulate liver injury responses. This study investigated the role of IL-6TS in the pathophysiology of AH. RNA sequencing of liver biopsies from patients with alcohol-related liver disease demonstrated a progressive decline in IL-6R expression correlating with increasing AH severity. Transforming growth factor (TGF)-β1 emerged as the most potent negative regulator of IL-6R expression. Notably, STAT3-dependent gene expression was increased in severe AH. *In vitro*, treatment of HepG2 cells with TGF-β1 suppressed IL-6R expression. Subsequent treatment with either IL-6 to stimulate classical signaling, or hyper–IL-6, a recombinant IL-6/IL-6R α peptide, to activate *trans*-signaling activated STAT3. Hyper–IL-6, but not IL-6, restored STAT3 activation in the face of suppressed IL-6R. RNA sequencing of hyper–IL-6 stimulated cells identified a gene signature that stratified a subset of AH patients with: i) enhanced IL-6TS activity, ii) increased intrahepatic neutrophilic infiltration, and iii) transcriptional enrichment of leukocyte migration pathways. Female mice treated with 10-day chronic-plus-binge ethanol exhibited enhanced STAT3 activation despite reduced hepatic IL-6R expression, leading to increased expression of neutrophilic activators, with colocalization of Ly6G^+^ leukocytes and STAT3^+^ hepatocytes. Collectively, these results indicate that IL-6TS preserves hepatocyte STAT3-dependent gene expression and promotes neutrophilic inflammation in AH.

## Graphical abstract

**Figure:**
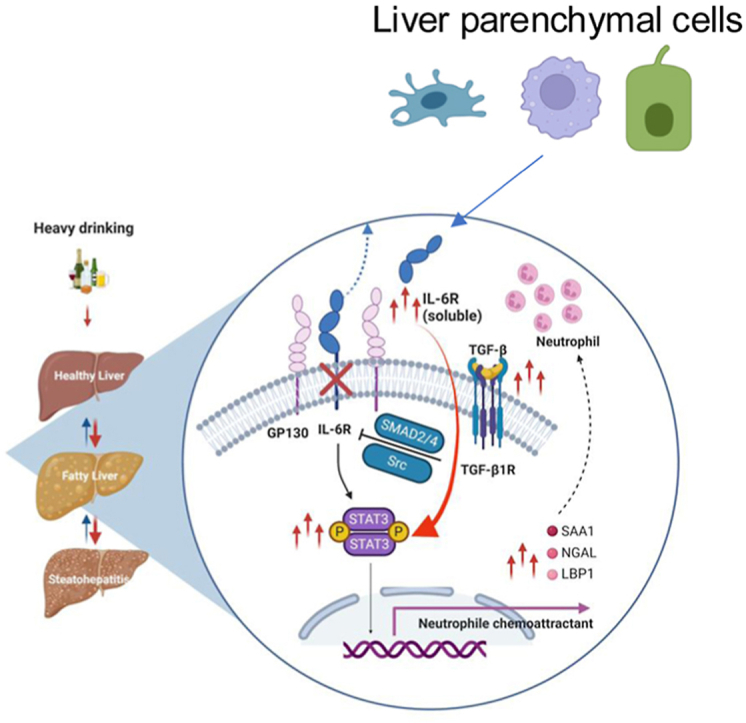

Alcohol-related liver disease (ALD) remains the leading cause of liver-related mortality in the United States,^1^ and its incidence is projected to increase in the coming decade in parallel with the rising prevalence of alcohol use disorder worldwide.^2^ In the United States, the rates of liver transplantation for ALD increased by 53% between 2017 and 2021, while those for hepatitis C virus–related disease declined by 62% over the same period.^3^ ALD encompasses a spectrum of conditions ranging from simple steatosis to fibrosis, cirrhosis, and hepatocellular carcinoma. Among these, alcohol-associated hepatitis (AH) is a form of acute-on-chronic liver failure characterized by systemic inflammation, multisystem organ failure, and a 1-month mortality rate approaching up to 30%.^4^ Despite the rapidly progressive clinical course of the disease, therapeutic options for AH remain limited to corticosteroids, which provide only modest short-term survival benefits.^5^

Neutrophilic inflammation is a key histologic feature of alcohol-related steatohepatitis (ASH), and multiple mechanisms contribute to neutrophil recruitment. First, alcohol-induced intestinal dysbiosis impairs intestinal barrier function to increase the translocation of pathogen-associated molecular patterns, including lipopolysaccharides (LPSs), into the liver.^6^, ^7^, ^8^ These pathogen-associated molecular patterns then activate macrophage/Kupffer cell inflammasomes via Toll-like receptors (TLRs), resulting in the elaboration of multiple proinflammatory cytokines and subsequent neutrophilic infiltration to perpetuate liver injury. Second, hepatocytes, either through oxidative stress or cytokine stimulation, release chemokines, most notably CXCL1 and IL-8, to drive neutrophilic inflammation.^9^, ^10^, ^11^ Murine studies have demonstrated that both neutrophil depletion and inhibition of neutrophil migration reduce alcohol-related liver injury, further underscoring the contribution of neutrophilic inflammation to ALD.^12^^,^^13^

IL-6 is a pleiotropic cytokine that exerts both pro- and anti-inflammatory effects. IL-6 mediates its biological effects by binding to its cognate receptor (IL-6R), which triggers the recruitment of the transmembrane signaling protein gp^130^, leading to the activation of STAT3. The IL-6/IL-6R/gp^130^ complex is required for signaling since IL-6 does not directly bind gp^130^. While gp^130^ is expressed on all human cells, membrane-bound (m) IL-6R is expressed in only the liver and some leukocyte subsets. Two distinct forms of IL-6 signaling have been characterized: classical signaling and *trans*-signaling. Signaling via mIL-6R is designated classical signaling and is limited to the liver and select leukocyte subsets. *Trans*-signaling involves the soluble form of IL-6R (sIL-6R), which can bind IL-6 and signal in cells that would otherwise not respond to IL-6 alone. Cells expressing mIL-6R are capable of activation via both pathways, and mIL-6R bioavailability is a key determinant of the balance between classical and *trans*-signaling in these cells.^14^

The role of IL-6 in AH is poorly understood. Increased serum IL-6 levels are associated with lower survival in AH.^15^^,^^16^ On the other hand, IL-6 is vital for liver homeostasis, as IL-6 both drives the acute phase response to infection and is necessary for hepatic regeneration after hepatectomy.^17^ One potential explanation for this paradoxical relationship is the relative balance between classical and *trans* IL-6 signaling in hepatocytes. In other systemic inflammatory disorders, IL-6TS enhances inflammatory activity in tissue-invasive myelocytes, endothelial cells, and hepatocytes.^18^, ^19^, ^20^, ^21^ Together, these findings point to a proinflammatory role for IL-6TS.^22^

The goal of this study was to dissect the role of IL-6TS in the pathophysiology of AH. The hypothesis tested was that IL-6TS results in STAT3 activation in hepatocytes, leading to the elaboration of neutrophilic activators driving neutrophilic inflammation.

## Materials and Methods

### Patients

**Figure 1:**
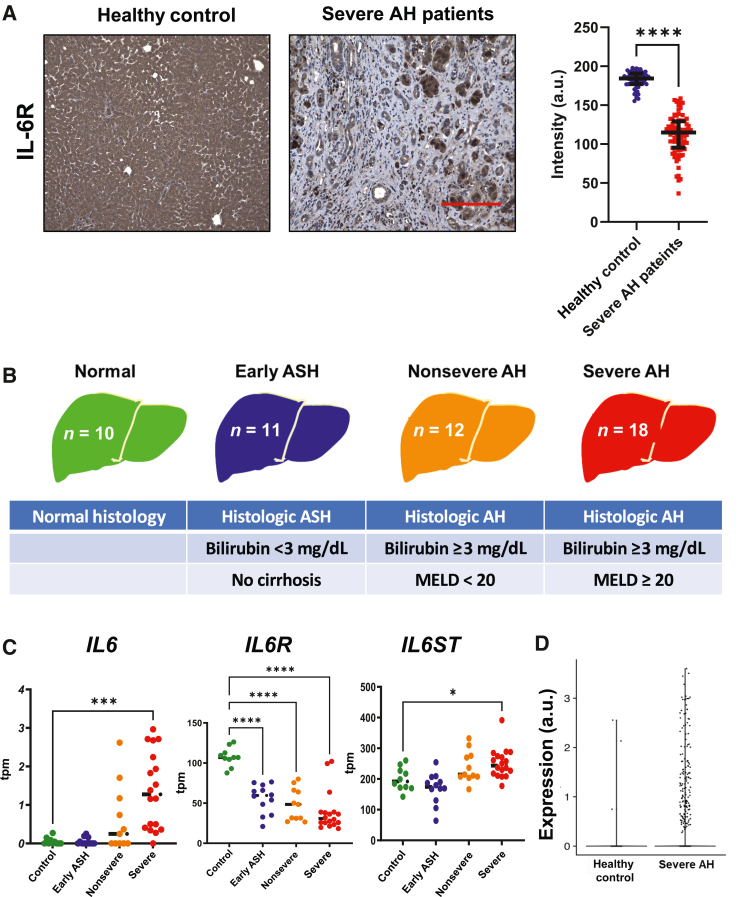
IL-6R expression is decreased in alcohol-associated hepatitis. **A:** Representative micrographs of liver biopsy specimens from a healthy control patient and a patient with alcohol-associated hepatitis (AH), stained with anti–IL-6R antibody; quantitative staining demonstrates that IL-6R is significantly reduced in livers of patients with alcohol-associated hepatitis versus health controls. Each data point represents signal intensity from a 40×-magnified section each of slide. **B:** Human phenotypes included in the InTeam RNA sequencing analysis for this study. **C:** Whole-liver mRNA expression in transcripts per million (tpm) from normal control livers, early alcohol-related steatohepatitis (ASH), nonsevere and severe AH livers of IL-6R, IL-6, and IL-6 signal transducer (IL-6ST). **D:** Hepatocyte single-cell IL-6R mRNA expression in raw counts from healthy controls and patients with severe AH. Individual data points are shown; bars represent median values. *n* = 3 subjects per group (**A**). ∗*P* < 0.05, ∗∗∗*P* < 0.001, and ∗∗∗∗*P* < 0.0001. *P =* 1.906 × 10^−241^ (**D**). Scale bar = 50 μm (**A**). a.u., arbitrary units.

Human liver samples were obtained from the Human Biorepository Core of the NIH-funded InTeam consortium (7U01AA021908-05), which has been previously described.^23^ All patients included gave written informed consent, and the research protocols were approved by the local ethics committees and by the central Institutional Review Board of the University of North Carolina (Chapel Hill, NC). The cohort consisted of 51 patients with varying ALD severity, including those with no liver disease (normal, *n* = 10), early ASH (*n* = 12), nonsevere AH (*n* = 11), and severe AH (*n* = 18). Patients with malignancies were excluded. The clinical characteristics of the patients are described in Figure 1B.

As a separate validation cohort, publicly available data sets of whole-liver RNA sequencing (RNA-seq) [severe AH (accession number GSE143318); *n* = 13; healthy controls, *n* = 7] and hepatocyte single-cell RNA-seq [severe AH (accession number GSE255772), *n* = 5; healthy controls (accession number GSE136103), *n* = 5] from patients with ALD were analyzed for targets of interest (GenBank; *https://www.ncbi.nlm.nih.gov/geo*).^24^^,^^25^

### HepG2 Cell Culture Experiments

HepG2 cells were obtained from ATCC (catalog number HB-8065, lot 70015966; Manassas, VA) and cultured in Dulbecco's modified Eagle's medium (catalog number 11965118), 10% fetal bovine serum (catalog number FB12999102; Fisher Scientific/Thermo Fisher Scientific, Waltham, MA), and 1% penicillin/streptomycin (catalog number 15140122; Gibco/Thermo Fisher Scientific, Schwerte, Germany). Cells were incubated at 37°C and 5% CO_2_. An equal number of cells were seeded in each well of either 6-well or 10-cm collagen-treated cell culture plates per treatment group and incubated overnight to attach, with subsequent media changes twice weekly.

HepG2 cells were pretreated with 1% fetal bovine serum with or without 5 ng/mL recombinant human transforming growth factor (TGF)-β1, catalog number R240-B-010; R&D Systems, Minneapolis, MN) overnight. After pretreatment, cells were treated for 1, 4, and 24 hours with either no treatment, recombinant human IL-6 protein 1 ng/mL (low dose), IL-6 20 ng/mL (high dose) (catalog number 206-IL-010; R&D Systems), hyper (h)–IL-6 [recombinant human IL-6R α/IL-6 chimera; hIL-6] 1 ng/mL (low dose), and hIL-6 20 ng/mL (high dose) (catalog number 8954-SR-025; R&D Systems). Untreated HepG2 control cells were washed twice with phosphate-buffered saline and provided with fresh medium. The following day, cells were used for protein and RNA isolation.

To assess the regulation of hepatocyte IL-6R expression *in vitro*, HepG2 cells were treated with TGF-β1 and cellular (c)-Src inhibitor PP2 (10 μmol/L; Calbiochem-EMD Millipore, Billerica, MA).

### Animal Experiments

Shp2 floxed (*Ptpn11* fl/fl) mice (gifted from F.G.H.) were maintained in a temperature-controlled facility under a 12-hour light/dark cycle with *ad libitum* access to food and water. For the ethanol studies, age-matched (14 to 16 weeks) female mice were subjected to the chronic–plus–single binge model; in a previous study, these mice were shown to exhibit a liver-injury response to alcohol ingestion similar to that in wild-type mice.^24^ Briefly, mice were acclimated to the Lieber-DeCarli control liquid diet (catalog number F1259SP; Bio-Serv, Flemington, NJ) for 5 days. Then, the mice were fed a 5% (v/v) ethanol liquid diet (ethanol; catalog number F1258SP; Bio-Serv) or pair-fed an isocaloric control liquid diet (pair) for 10 days. On day 11, mice received a single bolus of ethanol (5 g/kg body weight) or isocaloric maltose dextrin solution by oral gavage and sacrificed 9 hours later. The protocols of all mouse studies were approved by the Institutional Animal Care and Use Committee at UC Davis (Sacramento, CA).

### Silencing of SMAD4 in HepG2 Cells

SMAD4 siRNAs were purchased from Thermo Fisher Scientific (assay IDs s8403 and s8405). The effective working concentration of siRNA was 10 pmol/L in HepG2 cells, and siRNAs were transfected using Lipofectamine-RNAiMAX (InvitroGen/Thermo Fisher Scientific) following the manufacturer's recommendations. This protocol showed 70% to 85% of silencing efficiency (mRNA and protein levels) at 24 and/or 48 hours.

### Proteomics Analyses

Serum proteomics data were utilized from an observational study in patients with severe AH (*n* = 57), nonsevere AH (*n* = 17), and no liver disease (*n* = 16).^26^

### Genomic DNA Methylome Analysis

Genomic DNA (gDNA) methylome data derived from a previous study were used.^23^ Using the PureLink gDNA mini kit (Thermo Fisher Scientific), gDNA was extracted from flash-frozen tissue in six human livers explanted for AH and five healthy control livers, and quantified using the NanoDrop spectrophotometer (Thermo Fisher Scientific). As per the manufacturer's instructions, 1 μg of isolated gDNA was subjected to bisulfite conversion, denaturation, fragmentation, and hybridization to the Infinium MethylationEPIC BeadChip kit (Illumina, San Diego, CA). The BeadChips were scanned using an Illumina scan system, and intensity measurements were determined using iScan Control software version 4.0.0 (Illumina).

### ChIP-Seq of Histone Marks

Chromatin immunoprecipitation sequencing (ChIP-seq) data derived from a previous study were used.^27^ Briefly, liver tissue was obtained from six human livers explanted for AH, and four healthy control livers. Tissues were processed at the Mayo Clinic Center for Individualized Medicine Medical Genomics Facility (Rochester, MN) for RNA-seq and the Epigenomics Development Laboratory for ChIP-seq at the H3K27ac, H3K4me1, H3K4me3, and H3K27me3 marks, as previously described.^27^

### RNA Isolation and Quantitative Real-Time RT-PCR

Total RNA was extracted from HepG2cells and mouse liver using the Monarch Total RNA Miniprep kit (catalog number T2010S; New England Biolabs, Ipswich, MA) according to the manufacturer's instructions. Sample purity and concentration were measured by the NanoDrop spectrophotometer (Thermo Fisher Scientific).

### Quantitative Real-Time PCR

RNA isolation, cDNA generation, and quantitative real-time (q)PCR were performed as previously described.^27^^,^^28^ Total RNA from mouse liver (5 μg) and HepG2 (4 μg) were input to reverse transcribe with the SuperScript III First-Strand cDNA Synthesis kit (InvitroGen) according to manufacturer's instructions. Primers for mouse cDNA sequences were designed using Primer 3 version 0.4.0 (*http://bioinfo.ut.ee/primer3-0.4.0*). The amplification efficiency of all assays was calculated from the slope of a standard curve generated via 10-fold serial dilution of pooled control cDNA using the formula: Efficiency = 10 (–1/slope) – 1. The quantity and quality of the RNA were measured using NanoDrop. All samples were run in triplicate with SYBR Green master mix (Applied Biosystems/Thermo Fisher Scientific) on the ViiA 7 real-time PCR system (Applied Biosystems). The threshold cycle (C_T_) was determined by the ViiA 7 real-time PCR system, and the relative fold change of mRNA level was calculated using the ΔC_T_ method. Target gene expression was normalized with glyceraldehyde 3-phosphate dehydrogenase (GAPDH). The primer sequences used are listed in Tables 1 and 2.

**Table 1:** Human Oligonucleotide Sequences of Primers Used for Real-Time PCR

Gene	Full name	Primer	Sequence
*GAPDH*	Glyceraldehyde-3-phosphate dehydrogenase	Forward	5′-TACTAGCGGTTTTACGGGCG-3′
		Reverse	5′-TCGAACAGGAGGAGCAGAGAGCGA-3′
*HP*	Haptoglobin	Forward	5′-GACACCTGGTATGCGACTGGG-3′
		Reverse	5′-CTCCTGTCCACTCCCGTCCA-3′
*LBP*	Lipopolysaccharide binding protein	Forward	5′-CCTCTGCTCCGCTCCTGAAC-3′
		Reverse	5′-GCCACACTGAGCCGGAAGAC-3′
*LCN2*	Lipocalin 2	Forward	5′-TCCTCGGCCCTGAAATCATGC-3′
		Reverse	5′-CTTGCTCAGAGGTGGGGCTG-3′
*SAA1*	Serum amyloid A1	Forward	5′-TGGTTTTCTGCTCCTTGGTC-3′
		Reverse	5′-CCCGAGCATGGAAGTATTTG-3′
*SAA2*	Serum amyloid A2	Forward	5′-CCGAGCCCCATCAAAAGCCT-3′
		Reverse	5′-TAGCAGCCACCTCTCCCTGG-3′
*SOCS3*	Suppressor of cytokine signaling 3	Forward	5′-GGCCACTCTTCAGCATCTC-3′
		Reverse	5′-ATCGTACTGGTCCAGGAACTC-3′

**Table 2:** Mouse Oligonucleotide Sequences of Primers Used for Real Time PCR

Gene	Full name	Primer	Sequence
*Gapdh*	Glyceraldehyde-3-phosphate dehydrogenase	Forward	5′-GAAGCTTGTCATCAACGGGAAG-3′
		Reverse	5′-TTTGATGTTAGTGGGGTCTCGC-3′
*Icam1*	Intercellular adhesion molecule 1	Forward	5′-CCTGGAGACGCAGAGGACCT-3′
		Reverse	5′-ACGCCGCTCAGAAGAACCAC-3′
*Lbp*	Lipopolysaccharide binding protein	Forward	5′-GATCACCGACAAGGGCCTG-3′
		Reverse	5′-GGCTATGAAACTCGTACTGCC-3′
*Lcn2*	Lipocalin 2	Forward	5′-AATGTCACCTCCATCCTGGTC-3′
		Reverse	5′-GCCACTTGCACATTGTAGCTC-3′
*Junb*	Jun B proto-oncogene	Forward	5′-ACGACGACTCTTACGCAGCG-3′
		Reverse	5′-CAGGACCCTTGAGACCCCGA-3′
*Loxp4*	Lysyl oxidase-like 4	Forward	5′-TTCCAGGGCAAGGCAGTCAC-3′
		Reverse	5′-CACCGGGTTGACTGTCTGGG-3′
*Socs3*	Suppressor of cytokine signaling 3	Forward	5′-TCTTTGCCACCCACGGAACC-3′
		Reverse	5′-TGGAGGAGAGAGGTCGGCTC-3′

### Western Blot Analysis

HepG2 cells and mouse liver samples were homogenized on ice with the handheld Omni TH homogenizer (Omni International, Kennesaw, GA) with ice-cold RIPA lysis buffer containing Complete mini protease inhibitor cocktail and PhosSTOP phosphatase inhibitor cocktail (Roche Diagnostics, Manheim, Germany). In cell culture experiments, cells were lysed by adding ice-cold RIPA buffer directly to each well. Total lysates were collected by centrifuging for 20 minutes at 11,180 × *g* and 4°C. Then, protein concentration was determined with the Pierce BCA protein assay kit (Thermo Fisher Scientific) according to the manufacturer's protocol.

Equal amounts of protein (25 μg) were denatured in mPAGE 4XLDS sample buffer (MilliporeSigma, Burlington, MA) at 70°C for 10 minutes. Proteins were separated by SDS-PAGE and transferred onto a preactivated polyvinylidene difluoride membrane using the Trans-Blot Turbo transfer system (Bio-Rad Laboratories, Hercules, CA). Membranes were blocked with 5% nonfat milk in Tris-buffered saline with Tween-20 (TBST) for 2 hours, then probed with the desired primary antibodies overnight at 4°C. Membranes were washed in TBST, then incubated with the appropriate horseradish peroxidase–conjugated anti-rabbit or anti-mouse IgG secondary antibody for 1 hour at room temperature. Membranes were washed in TBST again and blots were developed using Clarity Western ECL substrate (Bio-Rad). Densitometry analyses were quantified by using Fujifilm Multi Gauge software version 3.0 (Fujifilm USA, Cambridge, MA) and normalized to β-actin. The antibodies used are listed in Table 3.

**Table 3:** Antibodies Used for Immunoblot Analysis

Antibody	MW	Supplier	Catalog no.	Dilution WB
Primary antibody				
pSTAT3	76	Cell Signaling Technology (Danvers, MA)	9145	1:2000
STAT3	86	Cell Signaling Technology	9139	1:1000
IL-6R	80	Proteintech (Rosemont, IL)	23457-1-AP	1:500
IL-6R	52	Abcam (Burlingame, CA)	Ab128008	1:500
gp^130^	17	Cell Signaling Technology	3732S	1:1000
β-Actin	42	Sigma-Aldrich (St. Louis, MO)	A5441	1:5000
Ly6G		Novus Biologicals (Centennial, CO)	NBP2-00441	1:300
MPO	59	Proteintech	22225-1-AP	1:300
Secondary antibody				
Anti-mouse		Jackson ImmunoResearch Laboratories (West Grove, PA)	115-035-003	1:10,000
Anti-rabbit		Jackson ImmunoResearch Laboratories	111-035003	1:10,000

MPO, myeloperoxidase; MW, molecular weight; WB, Western blot.

### IHC Analysis

Mouse livers were fixed in 4% paraformaldehyde for 48 hours at 4°C. Paraffin embedding, sectioning, and hematoxylin and eosin staining were performed by the UC Davis Center for Genomic Pathology laboratory. For hematoxylin and eosin staining, bright-field images were acquired by the Olympus BX51 microscope (Olympus, Center Valley, PA).

For immunofluorescence imaging of liver sections, deparaffinization and rehydration were done by incubating with xylene and ethanol solutions. Heat-mediated antigen retrieval was performed with IHC-Tek (catalog number IW-1100; IHC World, Ellicott City, MD), and samples were blocked by 1% bovine serum albumin at room temperature for 1 hour. Tissue sections were stained with anti–IL-6R (catalog number Ab128008; Abcam, Cambridge, MA), anti-pSTAT3 antibody (catalog number 9145; Cell Signaling Technology, Danvers, MA), anti-Ly6G (catalog number NBP2-00441; Novus Biologicals, Littleton, CO), anti-myeloperoxidase (MPO) (catalog number 2225-1-AP; Proteintech, Rosemont, IL) at 4°C overnight, followed with appropriate Alexa Fluor–conjugated secondary antibodies (catalog numbers Ab150160 and Ab150077; Abcam) at room temperature for 1 hour. Nuclei were counterstained with DAPI before the mounting of the coverslip (Vectashield mounting medium with DAPI; Thermo Scientific/Thermo Fisher Scientific). The slides were imaged using the 63× oil immersion objective fitted to the Zeiss LSM 700 confocal microscope (Carl Zeiss, Oberkochen, Germany), and images were processed using Zen software version 3.11 (Carl Zeiss). Whole-image optical density analysis was performed using ImageJ software version 1.51 (NIH, Bethesda, MD; *http://imagej.nih.gov.ij*).

### Immunofluorescence in HepG2 Cells

Cells were fixed with 3% formaldehyde in phosphate-buffered saline for 20 minutes, followed by incubation with blocking buffer [3% (w/v) bovine serum albumin, 5% (v/v) goat serum, and 0.1% Triton X-100 in phosphate-buffered saline] at room temperature for 1 hour. Cells were incubated with primary antibodies anti anti–IL-6R (catalog number Ab128008; Abcam) diluted at 2.5 μg/mL in blocking buffer overnight. After overnight incubation with primary antibodies at 4°C, the coverslips were stained with goat anti-rabbit Alexa Fluor 488 (catalog number A11008; Thermo Scientific) diluted in blocking buffer at 1:3000. Nuclei were counterstained with DAPI (diluted 1:200) before the mounting of the coverslip (Vectashield mounting medium with DAPI). Slides were then visualized using confocal microscopy, and optical densities were quantified as described in the previous paragraph.

### RNA Sequencing Analysis

RNA-seq library production and sequencing were performed by Novogene Corporation, Inc. (Sacramento, CA). RNA degradation and contamination were monitored on 1% agarose gels, and purity was checked using the NanoPhotometer spectrophotometer (Implen GmbH, Munich, Germany). RNA integrity and quantitation were assessed using the RNA Nano 6000 assay kit of the Agilent Bioanalyzer 2100 system (Agilent, Santa Clara, CA). A total of 1 μg RNA per sample was used as input material. Sequencing libraries were generated using the NEBNext Ultra RNA library prep kit for Illumina (New England Biolabs) following the manufacturer's recommendations, and index codes were added to attribute sequences to each sample. Briefly, mRNA was purified from total RNA using poly-T oligo-attached magnetic beads. Fragmentation was performed using divalent cations under elevated temperature in NEBNext RNA First Strand synthesis reaction buffer. First-strand cDNA was synthesized using random hexamer primers and M-MuLV reverse transcriptase (RNase H). Second-strand cDNA synthesis was subsequently performed using DNA polymerase I and RNase H. Remaining overhangs were converted into blunt ends via exonuclease/polymerase activities. After adenylation of 3′ ends of DNA fragments, NEBNext adaptors with hairpin loop structure were ligated to prepare for hybridization. To preferentially select cDNA fragments of approximately 150 to 200 bp in length, the library fragments were purified with the AMPure XP system (Beckman Coulter, Brea, CA). Three microliters of USER Enzyme (New England Biolabs) was used with size-selected, adaptor-ligated cDNA at 37°C for 15 minutes followed by 5 minutes at 95°C before PCR. PCR was performed with Phusion High-Fidelity DNA polymerase, NEBNext Universal PCR primer for Illumina, and NEBNext Index primer (New England Biolabs). PCR products were purified again with the AMPure XP system, and library quality was assessed on the Agilent Bioanalyzer 2100. The clustering of the index-coded samples was performed on a cBot Cluster Generation System using the PE Cluster Kit cBot-HS (Illumina) according to the manufacturer's instructions. After cluster generation, the library preparations were sequenced on an Illumina NovaSeq S4 platform and 150-bp paired-end reads were generated. Raw reads (FASTQ) were processed through fastp software version 0.20.0 (*https://hpc.nih.gov/apps/fastp.html*), removing reads containing adapter and poly-N sequences and low-quality reads while simultaneously calculating Q20, Q30, and GC content. Paired-end clean reads were aligned to the mouse reference genome (grcm38) using the Spliced Transcripts Alignment to a Reference (STAR) software version 2.6.1d (*https://github.com/alexdobin/STAR/releases*). FeatureCounts software version 1.5.0-p3 (*https://subread.sourceforge.net*) was used to count the reads mapped to each gene; the reads per kilobase per million mapped reads (RPKM) of each gene was calculated based on the length of the gene and reads mapped to the gene. Differentially expressed genes were then analyzed with Ingenuity Pathway Analysis (Qiagen, Hilden, Germany) to identify upstream regulators of the observed transcriptional profile, and the Ingenuity Pathway Analysis disease analysis tool was used for pathway analysis of gene networks.

### Enzyme-Linked Immunosorbent Assay

To investigate the acute phase response by serum amyloid A (SAA)-1, factor VII, and lipocalin (LCN)-2, cell culture supernatant levels with or without TGF-β1–conditioned IL-6 and hIL-6 treatment were measured by enzyme-linked immunosorbent assay [human SAA kit (SAA1; catalog number ab100635; Abcam); human factor VII kit (catalog number ab190810; Abcam); and human LCN-2/neutrophil gelatinase-associated lipocalin (NGAL; Quantikine kit; catalog number DLCN20; R&D Systems, Inc., Minneapolis, MN)] as per the manufacturers' instructions. In short, cell culture supernatant was added to the plate and incubated for 2 hours at room temperature. After the wash, a detection antibody was added and incubated for 2 hours. The plate was washed, and streptavidin–horseradish peroxidase was added followed by incubation for 20 minutes. The plate was then washed a final time before a substrate solution was added before 20 minutes of incubation. At the end of the incubation, stop solution was added and absorbance was measured at 450 nm using the BioTek Synergy H1 microplate reader (Agilent).

### Hierarchical Clustering

Generalized linear models of human *IL6R* transcript abundance were generated from human RNA-seq data using both transcript data and alcohol-related liver disease severity as covariates. Hierarchical clustering of covariate coefficients was performed using average linkage and the Ward method in R software package version 4.1.2 (*http://www.r-project.org*). Results were depicted as a dendrogram with heatmap ranging from –3 (green) to 0 (black) to +3 (red). Disease severity cohorts included healthy controls (1), early ASH (2), compensated alcohol-related cirrhosis (3), nonsevere AH (4), and severe AH (5), as noted in Figure 1B.

In the human RNA-seq cohorts, hierarchical clustering analysis was performed with the IL-6TS gene signature derived from HepG2 cultures using the Interactive Cluster Heat Map Builder (*https://build.ngchm.net/NGCHM-web-builder*, last accessed February 17, 2025) with average linkage and the Ward method. Human data (in transcripts per million; tpm) were normalized to means of 0 and variance ranging from 0 to 1.

### Data Availability

The HepG2 RNA-seq data reported in this article have been deposited in the Gene Expression Omnibus database (*https://www.ncbi.nlm.nih.gov*; accession number GSE255379).

### Statistical Analysis

For RNA sequencing, differential expression analysis between two groups (three biological replicates per group) was performed using DESeq2 R shareware package version 1.20.0 (*https://bioconductor.org/packages/devel/bioc/html/DESeq2.html*). The resulting *P* values were adjusted using the Benjamini and Hochberg approach to control the false discovery rate. Genes with an adjusted *P* value of <0.05 by DESeq2 were considered differentially expressed. The rank sum test was used to compare differences in continuous variables between two groups. For multiple group comparisons, including continuous clinical data and tpm counts, the Kruskal-Wallis test followed by the uncorrected Dunn test was performed with Prism software version 9.0 (GraphPad Software, San Diego, CA). Data are presented as medians with 25th to 75th percentile interquartile ranges (IQRs). Categorical variables were compared between groups using the Fisher exact test, and *P* < 0.05 was considered statistically significant. Spearman correlation was used to assess relationships between RNA-seq data expressed as tpm.

## Results

### TGF-β1 Suppresses Hepatic IL-6R Expression in Severe AH

As mIL-6R is a key determinant of the relative balance between *trans* and classical signaling in hepatocytes,^29^ IL-6R expression in healthy human and severe AH livers was assessed by immunohistochemistry. In severe AH specimens, there was reduced staining in hepatocytes compared to that in controls, and most IL-6R staining was localized to areas of ductular reaction (Figure 1A). RNA-seq data from the InTeam Consortium's Human Biorepository were used to determine *IL6R* transcript abundance in ALD. This cohort consisted of 51 patients with varying ALD severity, including those with no liver disease (normal, *n* = 10), early ASH (*n* = 12), nonsevere AH (*n* = 11), and severe AH (*n* = 18) (Figure 1B). Although the expression of IL-6 and IL-6 signal transducer (IL-6ST)/gp^130^ was significantly increased with more severe disease, hepatic *IL6R* expression was markedly reduced in severe AH (Figure 1C).

Analysis of an independent, publicly available single-cell hepatocyte RNA (scRNA) data set demonstrated increased hepatocyte *IL6R* expression in AH compared to that in healthy controls^25^ (Figure 1D). The metalloproteinases a disintegrin and metalloproteinase domain-containing protein (ADAM)-10 and ADAM17 regulate sIL-6R expression.^30^ N-glycosylation via oligosaccharyltransferase (OST) is crucial to protein folding and localization of IL-6R.^31^^,^^32^ Ethanol inhibits N-glycosylation by depleting dolichol phosphate, a key precursor N-glycan synthesis, which leads to under-glycosylation of target proteins.^33^ Together, these findings suggest that post-translation modification, protein degradation, and/or translational inhibition may also contribute to IL-6R expression in AH.

**Figure 2:**
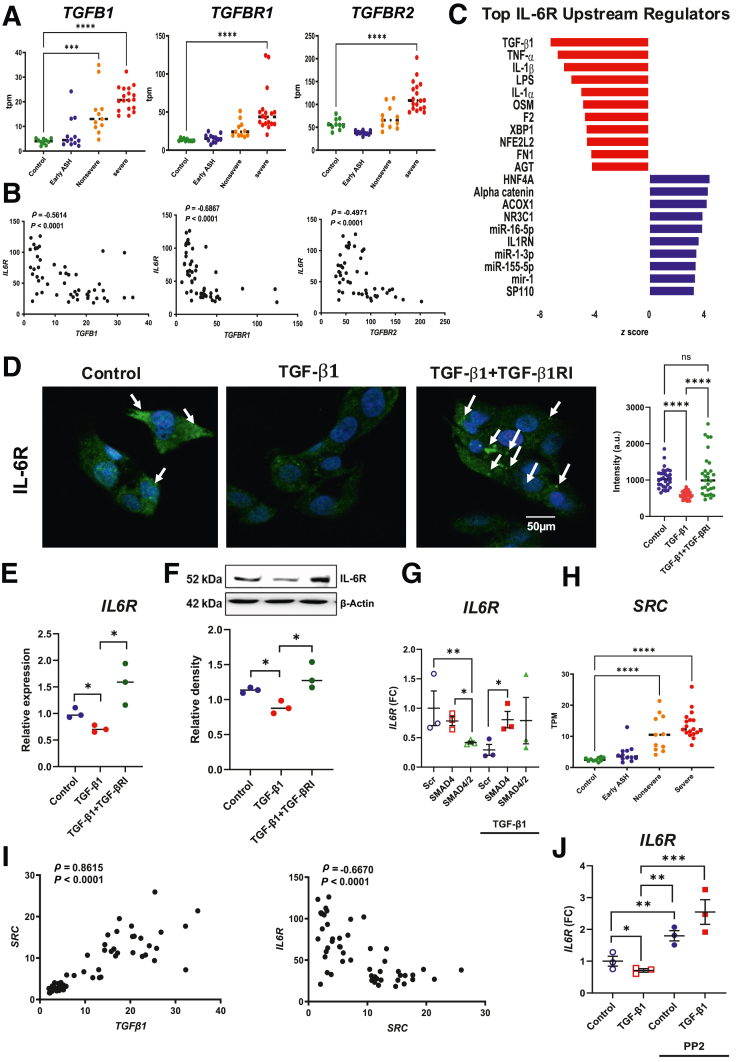
TGF-β1 suppresses hepatic IL-6R expression in alcohol-associated hepatitis (AH). **A:** mRNA abundance in transcripts per million (tpm) from normal control livers, early alcohol-related steatohepatitis (ASH), nonsevere and severe AH livers of TGF-β1, TGF-βR1 inhibitor SB431542 (TGF-βRI), and TGF-βRII. **B:** Spearman rank correlation between mRNA levels of IL-6R and TGF-β1, TGF-βRI, and TGF-βRII. **C:** Ingenuity Pathway Analysis identified TGF-β1 as the strongest negative regulator of IL-6R expression in human AH. **D:** HepG2 cells were treated either 1% fetal bovine serum control, TGF-β1 for 4 hours, or TGF-β1 for 4 hours after pretreatment with TGF-βRI (5 nmol/L); representative immunofluorescence micrographs of treated HepG2 cells after staining with anti–IL-6R antibody are shown. **Arrows** indicate immunostaining of IL-6R. **E** and **F:** Quantitative real-time (q)-PCR of *IL6R* (**E**) and immunoblot analysis of IL-6R protein (**F**) in HepG2 cells further confirm that down-regulation of IL-6R expression by TGF-β1 is inhibited by blocking TGF-βRI. **G:** SMAD4-silenced HepG2 cells were treated overnight with TGF-β1 for 8 hours (qPCR of *IL6R* mRNA is shown). **H:** mRNA abundance (in tpm) from normal control mRNA abundance (in tpm) from normal control livers, early ASH, nonsevere and severe AH livers of Src. **I:** Spearman rank correlation between mRNA levels of Src and TGF-β1 and IL-6R are shown. **J:** HepG2 cells were treated with TGF-β1 overnight in the presence of c-Src inhibitor PP2 (10 μmol/L); qPCR of *IL6R* mRNA is shown. Individual data points are shown, and bars represent median values (**A** and **H**). *n* = 3 (**G**). ∗*P* < 0.05, ∗∗*P* < 0.01, ∗∗∗*P* < 0.001, and ∗∗∗∗*P* < 0.0001. Scale bar = 50 μm (**D**). ns, not significant.

Hierarchical clustering showed that IL-6R expression was dependent on ALD severity (Supplemental Figure S1), and hence, disease severity was incorporated into generalized linear models of RNA-seq data to determine key upstream regulators of IL-6R expression. Among 3513 genes, TGF-β1 was the most potent negative regulator of IL-6R expression in severe AH (Figure 2C). Furthermore, mRNA expression levels of *TGFB1* and its receptors (*TGFBR1* and *TGFBR2*) were markedly increased in AH livers and exhibited strongly negative correlations with *IL6R* mRNA (Figure 2, A and B). *In vitro*, IL-6R expression was measured in HepG2 cells using immunocytochemistry, Western blot analysis, and qPCR; TGF-β1 treatment suppressed IL-6R expression, and the effect was reversed by the TGF-βRI inhibitor SB431542 (Figure 2, D–F). Furthermore, TGF-β1 decreased *IL6R* mRNA levels in HepG2 cells, while the inhibition of nuclear translocation of SMAD family proteins via SMAD4 knockdown restored *IL6R* expression *in vitro* (Figure 2G), indicating that TGF-β1 suppresses hepatic IL-6R expression in AH in a SMAD-dependent manner. Prior work has also shown that c-Src mediates TGF-β1 signaling in AH.^23^ In the InTeam cohort, *c-SRC* was significantly upregulated in livers from patients with AH and demonstrated a strong positive correlation with TGF-β1 levels (Figure 2, H and I). As with TGF-β1, hepatic *c-SRC* expression was negatively correlated with *IL6R* (Figure 2I), and *in vitro*, TGF-β1–mediated suppression of IL-6R expression in HepG2 cells was reversed after treatment with the c-Src inhibitor PP2 (Figure 2J). Together, these findings implicate both SMAD- and c-Src–dependent suppression of *IL6R* expression by TGF-β1.

As TGF-β1 exposure drives epigenetic modifications in both hepatocytes and cholangiocytes in chronic liver disease,^23^^,^^34^ the methylation status of *IL6R* gene loci was analyzed in normal (*n* = 5) and AH (*n* = 6) livers, and no significant differences between groups was found (Supplemental Figure S2A). Data from H3K27Ac, HK27me3, H3K4me1, and H3K4me3 ChIP-seq of normal (*n* = 5) and AH (*n* = 8) livers demonstrated no significant differences in chromatin structure at either IL-6R loci or IL-6R promoter binding sites (Supplemental Figure S2B), suggesting that TGF-β1 suppression of IL-6R in AH is not due to epigenetic modification. Instead, direct transcriptional inhibition is likely the predominant mechanism of IL-6R regulation by TGF-β1.

### Severe AH Is Characterized by Enhanced STAT3 Activation despite Reduced IL-6R Expression

**Figure 3:**
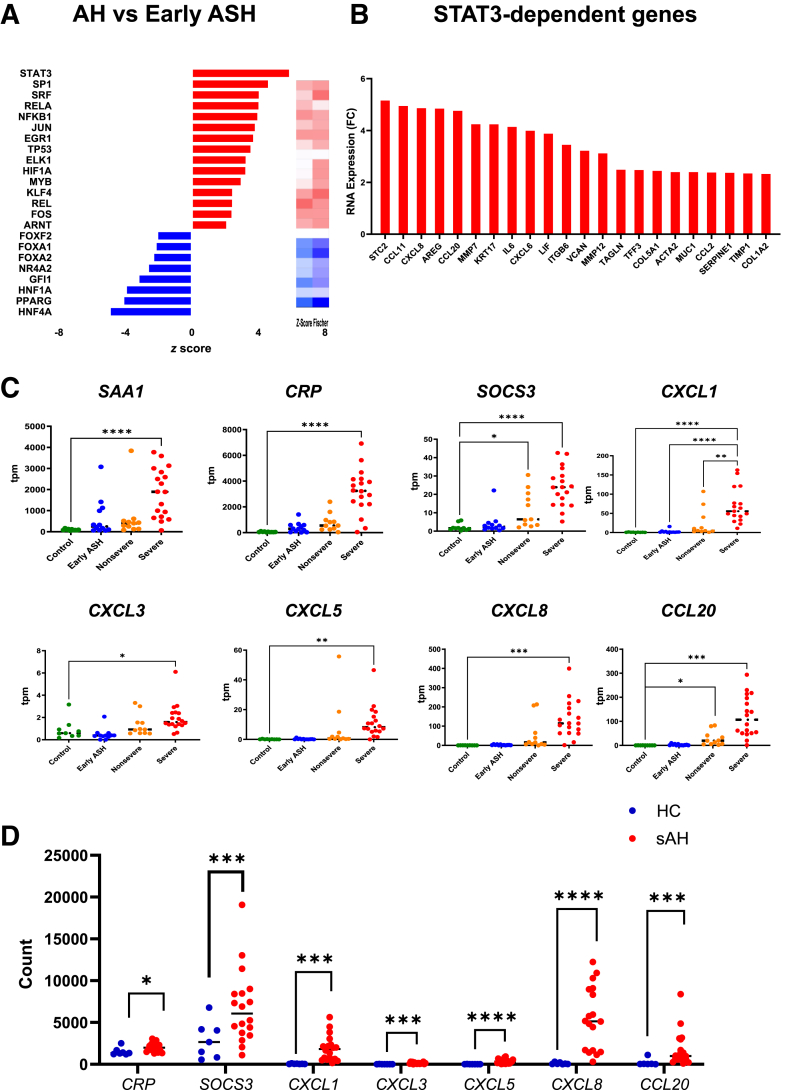
STAT3 activation persists in AH despite reduced IL-6R expression. **A:** Transcription factor transcriptomic footprint inferred from ingenuity pathway analysis and oPOSSUM analysis between early alcohol-related steatohepatitis (ASH) and alcohol-associated hepatitis (AH) identifies STAT3 as the most strongly enhanced liver-enriched transcription factor. **B:** Top, differentially expressed genes comprising the STAT3 activation profile are shown as fold changes between AH an ASH. All genes had false discovery rate of <10^−6^. **C:** mRNA abundance [in transcripts per million (tpm)] from normal control livers, early ASH, nonsevere and severe AH livers of *SAA1*, *CRP*, *SOCS3*, *CXCL1*, *CXCL3*, *CXCL5*, *CXCL8*, and *CXCL20*. **D:** mRNA abundance in raw counts of target genes from an independent cohort of healthy control (HC) and severe AH (sAH) livers. *n* = 7 (**D**, HC); *n* = 13 (**D**, sAH). ∗*P* < 0.05, ∗∗*P* < 0.01, *∗∗∗P* < 0.001, and ∗∗∗∗*P* < 0.0001.

InTeam liver RNA-seq data were examined to assess the predicted activity of hepatic transcription factors in AH. The search of transcription factor motifs of differentially expressed genes was combined with ingenuity pathway analysis as previously described.^23^ It indicated that AH was associated with a profound increase in the activity of STAT3, the canonical transcription factor of IL-6 activation (Figure 3A); the top differentially expressed genes comprising the STAT3 activation profile are shown in Figure 3B. These encompassed genes for crucial fibrogenesis and stellate cell activation (*COL5A1*, *COL1A2*, *MMP7*, *MMP12*, *ACTA2*, *VCAN, TIMP1*, and *TAGLN*), duct reaction (*KRT17*, *ITGB6*, *TFF3*, and *MUC1*), inflammatory cytokines and associated acute phase reactants (*IL6*, *LIF,* and *SERPINE1*), and neutrophil chemokines (*CCL11*, *CXCL8*, *CCL20*, *CXCL6*, and *CCL2*). Although hepatic IL-6R expression is markedly reduced with increasing AH severity, many hepatic STAT3-dependent genes were up-regulated in severe AH, including acute phase reactants, multiple neutrophil chemokines, and *SOCS3*, the primary negative regulator of STAT3 phosphorylation (Figure 3C). These findings were validated in an independent whole-liver RNA-seq data set that included 13 patients with AH and 7 healthy controls^25^ (Figure 3D).

**Figure 4:**
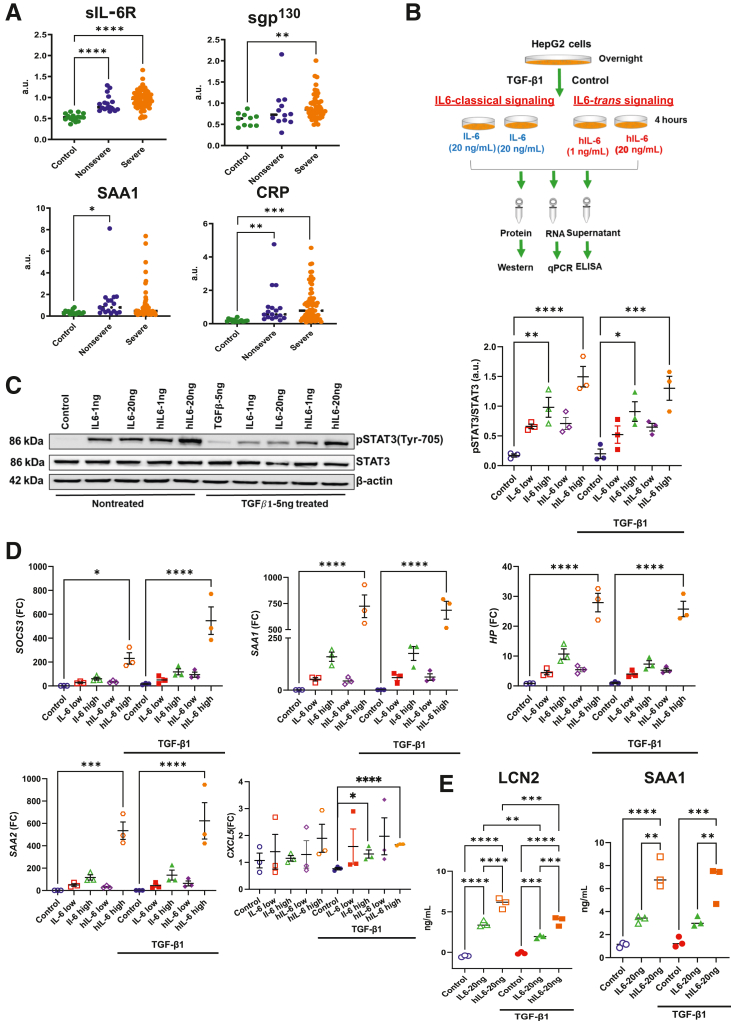
IL-6 *trans*-signaling can overcome TGF-β1–mediated IL-6R suppression to restore STAT3 activation in hepatocytes. **A:** Serum proteomics quantification of circulating levels of sIL-6R, sgp^130^, serum amyloid A (SAA)-1 and C-reactive protein (CRP) from patients with normal liver function, nonsevere alcohol-associated hepatitis (AH), and severe AH. **B:** HepG2 cells were pretreated overnight with either TGF-β1 or 1% fetal bovine serum control, followed by treatment with either IL-6 or hyper (h)–IL-6 at low (1 ng/mL) or high (20 ng/mL) doses. **C:** Immunoblot analysis of pSTAT3Y705 and STAT3 expression in treated HepG2 cells with quantification of immunoblot signal intensity. **D:** Quantitative real-time PCR mRNA expression of *SOCS3*, *HP*, *SAA1*, *SAA2*, and *CXCL5* in treated HepG2 cells. **E:** Supernatants were collected from treated HepG2 cells for protein quantification by enzyme-linked immunosorbent assay of SAA1 and neutrophil gelatinase-associated lipocalin (NGAL). ∗*P* < 0.05, ∗∗*P* < 0.01, ∗∗∗*P* < 0.001, and ∗∗∗∗*P* < 0.0001.

Since STAT3 activation is increased in AH despite TGF-β1–mediated suppression of IL-6R, it was hypothesized that IL-6TS can overcome TGF-β1–mediated IL-6R suppression to restore STAT3 activation. To test this hypothesis, serum proteomics data were analyzed from an observational study of patients with severe AH (*n* = 57), nonsevere AH (*n* = 17), and no liver disease (*n* = 16).^26^ Patients with severe AH demonstrated elevated relative circulating levels of sIL-6R, sgp^130^, and multiple STAT3-dependent acute phase reactants, including C-reactive protein (CRP) and SAA1 (Figure 4A), despite reduced hepatic mIL-6R expression. In HepG2 cells, treatment with both IL-6 and hIL-6 stimulated dose-dependent STAT3 phosphorylation which was suppressed by TGF-β1. However, only high-dose hIL-6 (20 ng/mL) restored STAT3 phosphorylation, while TGF-β1–mediated inhibition of STAT3 persisted despite high-dose IL-6 (20 ng/mL) treatment (Figure 4, B and C). Corresponding changes were observed in STAT3-dependent gene expression, as high-dose hIL-6 treatment of TGF-β1–conditioned cells enhanced gene expression of *SOCS3* and of multiple acute phase reactants, including *SAA1, SAA2*, and *HP* accompanied by enhanced secretion of SAA-1 and NGAL (Figure 4, D and E). Among direct neutrophil chemokines, *CXCL5* expression was increased with hIL-6 stimulation of TGF-β1–conditioned HepG2 cells (Figure 4D).

### IL-6TS Generates a Specific Gene Expression Profile in HepG2 Cell Lines

**Figure 5:**
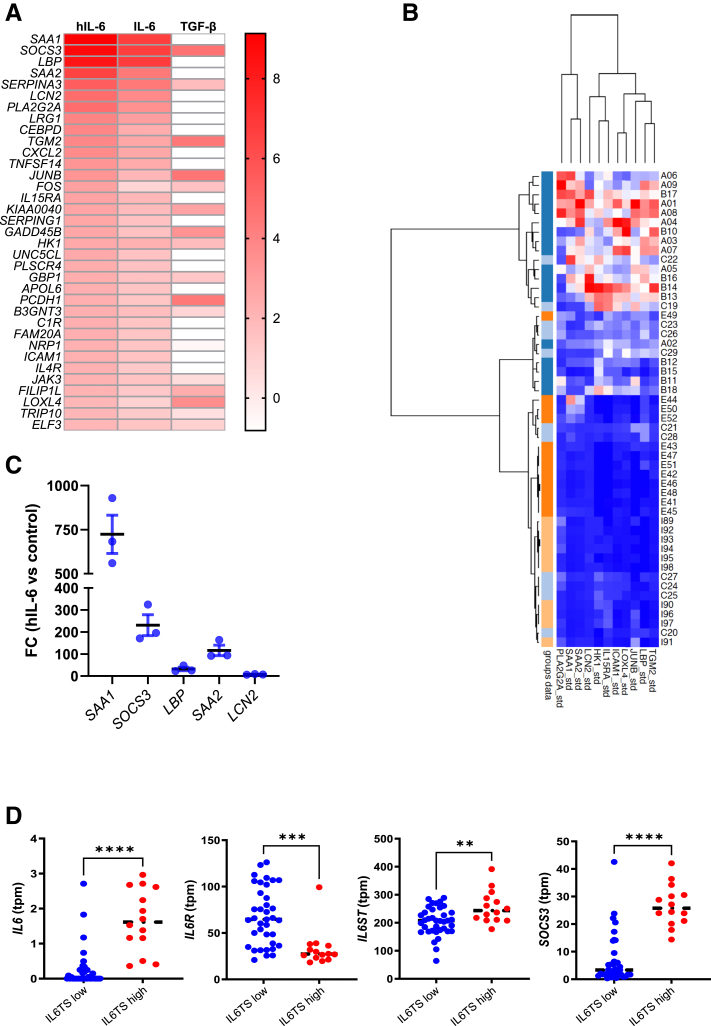
Trans IL-6 signaling induces a specific gene expression profile in HepG2 cells. HepG2 cells were stimulated with either 1% fetal bovine serum (control) IL-6 (20 ng/mL), hyper (h)–IL-6 (20 ng/mL), or TGF-β1 (5 ng/mL). Gene expression was quantified by RNA sequencing, normalized, and compared with nonstimulated controls. **A:** The most up-regulated genes by hIL-6 are shown as heat map of log_2_ fold change. **B:** Quantitative real-time PCR of top differentially expressed genes in hyper (h)–IL-6-stimulated HepG2 cells. **C:** Hierarchical clustering of whole liver transcriptomic data from InTeam cohort patients using the IL-6TS–specific gene signature. **D:** mRNA abundance in transcripts per million (tpm) from IL-6TS–high and IL-6TS–low livers of IL6 signal transducer (IL-6ST), IL6, IL6R, and suppressor of cytokine signaling (SOCS)-3. *n* = 51 (**C**). ∗∗*P* < 0.01, ∗∗∗*P* < 0.001, and ∗∗∗∗*P* < 0.0001. All, *P* < 0.001 (**B**).

To characterize a hepatocyte gene signature regulated by IL-6TS, HepG2 cells were stimulated with hIL-6 (20 ng/mL) or IL-6 (20 ng/mL), and global gene expression was analyzed via RNA-seq. A total of 2211 protein-coding genes (*q* < 0.05) were differentially expressed between hIL-6–stimulated and –nonstimulated cells, and 398 genes were differentially expressed with IL-6 (20 ng/mL) alone. A total of 390 genes overlapped between hIL-6 and IL-6, with a consistent directional change in expression, with hIL-6 eliciting more pronounced absolute changes than IL-6 (Figure 5A). Several of the genes most strongly up-regulated by IL-6TS were acute phase reactants implicated in myeloid cell migration and activation (*SAA1*, *SAA2*, *LCN2*, *LBP, TNFSF14, CXCL2,* and *ICAM1*), and genes associated with complement activation (*C1R* and *SERPING1*), hepatic lipid metabolism (*LRG1*, *APOA6,* and *HK1*), regulation of cell death or differentiation (*FOS*, *JUN*, *TGM2*, *ICAM1*, and *NRP1*), and janus tyrosine kinase (JAK)/STAT signaling (*SOCS3* and *JAK3)*. qPCR was used to confirm up-regulation of the top IL-6TS–related genes in HepG2 cells (Figure 5C). As a separate control, HepG2 cells were stimulated with TGF-β1 (5 ng/mL), since TGF-β1 is an established driver of defective hepatocyte nuclear factor (HNF)-4α–dependent gene expression, transcriptional reprogramming, and hepatocellular failure in AH.^23^ The TGF-β1 gene expression signature was distinct from both IL-6TS and IL-6 gene profiles, as TGF-β1 had minimal effect on most IL-6TS–specific genes. Conversely, IL-6TS had no effect on the most frequent TGF-β1–stimulated genes in HepG2 cells.

To further validate this gene signature, experiments were repeated in HepG2 cells preconditioned overnight with TGF-β1. Fifty-eight genes were up-regulated in TGF-β1+hIL-6 versus TGF-β1 cells (*q* < 0.05; log_2_ fold change, ≥2.0), and 22 genes were up-regulated in TGF-β1+IL-6 cells (*q* < 0.05; log_2_ fold change, ≥2.0). All TGF-β1+IL-6–up-regulated genes were also increased with TGF-β1+hIL-6. The most frequent differentially expressed genes, *SAA1*, *LBP*, *SAA2*, and *LCN2,* were identical to those identified in hIL-6–treated HepG2 cells in the absence of TGF-β1 preconditioning (Supplemental Figure S3).

To determine whether the IL-6TS pathway is activated in samples of human livers with ALD, hierarchical clustering analysis was conducted on genes most strongly up-regulated by IL-6 *trans*-signaling in HepG2 cultures. A set of 11 co-clustered genes (*SAA1*, *SAA2*, *LCN2*, *LBP*, *ICAM1*, *LOXL4*, *PLA2G2A*, *HK1*, *JUNB*, *TGM2,* and *IL15RA*) were identified that characterized a subset of AH patients from the InTeam with enhanced expression of these IL-6TS–inducible transcripts (referred to as IL-6TS^+^) (Figure 5B). The IL-6TS^+^ subset exhibited significant up-regulation in IL-6/JAK/STAT3-dependent genes despite marked reductions in *IL6R* expression (Figure 5D). To further validate this gene signature, additional hierarchical clustering analyses were performed on an independent whole-liver RNA-seq data set consisting of 13 patients with AH and 7 healthy controls^25^ (Gene Express Omnibus, *https://www.ncbi.nlm.nih.gov/geo*; accession number GSE 143318) (Supplemental Figure S4). Together, these findings highlight a novel gene signature that identifies a subset of patients with ALD with enhanced IL-6TS–dependent STAT3 activation in the face of reduced mIL-6R bioavailability.

### IL-6TS^+^ Subset Is Characterized by More Severe Liver Dysfunction, Portal Hypertension, and Neutrophilia

Clinical and histologic features of patients stratified by IL-6TS status are summarized in Table 4. No significant differences were observed in the two subsets (IL-6TS^+^ and IL-6TS^–^) in terms of sex, renal function, or alanine aminotransferase (ALT) levels. Serum biochemistry analysis revealed that the IL-6TS^+^ subset exhibited more pronounced hepatocellular and cholestatic liver injury as compared to the IL-6TS^–^ subset, as evidenced by elevated aspartate aminotransferase (AST) and γ-glutamyl transferase (GGT) levels, respectively. Indicators of liver dysfunction, including serum bilirubin, international normalized ratio, and Model for End-Stage Liver Disease (MELD) score, were also significantly higher in the IL-6TS^+^ cohort. Markers of portal hypertension, such as hepatic venous pressure gradient and platelet count, were more pronounced in the IL-6TS^+^ cohort compared to the IL-6TS^–^ cohort, despite comparable fibrosis severity in patients with available liver biopsies (*n* = 35).

**Table 4:** Clinical, Laboratory and Liver Histologic examination Features in IL-6TS^+^ and IL-6TS^–^ Patients with Alcohol-Associated Liver Disease

Feature	Value	IL-6TS^+^ (*n* = 14)	IL-6TS^−^ (*n* = 37)	*P*
Age, years	49 (41 to 55)	54 (47 to 58)	48 (39 to 53)	0.0494
Female sex, *n* (%)	19 (37.3)	5 (37.3)	14 (37.8)	0.578
HVPG, mmHg	19 (14 to 22)	22 (19 to 23)	14 (9 to 19)	0.0035
Hemoglobin, g/dL	13.2 (11.3 to 14.4)	11.6 (11.1 to 12.8)	13.8 (11.8 to 14.8)	0.0037
WBC, × 10^9^/L	7.5 (5.5 to 11.5)	8.8 (7.8 to 11.9)	6.5 (5.0 to 7.8)	0.0154
Platelets, × 10^9^/L	181 (90 to 236)	124 (67 to 209)	196 (117 to 237)	0.0358
AST, IU/L	112 (48 to 160)	161 (117 to 240)	72 (28 to 125)	0.0009
ALT, IU/L	45 (32 to 67)	56 (32 to 67)	43 (26 to 64)	0.5544
GGT, IU/L	269 (77 to 604)	414 (189 to 687)	186 (27 to 475)	0.0575
Bilirubin, mg/dL	1.5 (0.7 to 13.7)	13.2 (10.0 to 20.5)	1.0 (0.7 to 1.7)	0.0001
INR, a.u.	1.2 (1.0 to 1.6)	1.6 (1.4 to 1.7)	1.0 (1.0 to 1.3)	0.0002
Albumin, g/dL	3.5 (2.6 to 4.5)	2.5 (2.3 to 2.9)	4.1 (3.2 to 4.6)	0.0001
Creatinine, mg/dL	0.77 (0.60 to 0.96)	0.73 (0.60 to 1.16)	0.81 (0.61 to 0.94)	0.9831
Sodium, mmol/L	137 (134 to 140)	134 (132 to 137)	139 (136 to 140)	0.0109
Child score	9 (8 to 11)	11 (9 to 11)	8 (6 to 9)	0.0031
MELD score	22 (14 to 24)	24 (21 to 26)	17 (11 to 22)	0.0379
ABIC score	7.5 (7.1 to 8.9)	8.3 (7.1 to 8.9)	6.7 (6.0 to 8.0)	0.0219
Histologic examination, *n* (%)				
Fibrosis				0.175
None/portal	13/35 (37.2)	2/10 (20.0)	11/25 (44.0)	
Bridging/cirrhosis	22/35 (62.8)	8/10 (80.0)	14/25 (56.0)	
Neutrophilia	6/35 (17.1)	5/10 (50.0)	1/25 (4.0)	0.004
Bilirubinostasis				0.008
None	23 (65.7)	4/10 (40.0)	19/25 (76.0)	
Hepatocellular	3/35 (8.6)	2/10 (20.0	1/25 (4.0)	
Canalicular/ductular	4/35 (11.4)	0/10 (0.0)	4/25 (16.0)	
Mixed hepatocellular and canalicular/ductular	5 (14.3)	4/10 (40.0)	1/25 (4.0)	

All continuous variables are expressed as medians with interquartile range.

ABIC, age bilirubin INR creatinine score; ALT, alanine aminotransferase; AST aspartate aminotransferase; a.u., arbitrary units; GGT, γ-glutamyl transferase; HVPG, hepatic venous pressure gradient; INR, international normalized ratio; MELD, Model for End-Stage Liver Disease; WBC, white blood cell.

The IL-6TS^+^ cohort exhibited more significant systemic inflammation as circulating white blood cell counts were increased. Hepatic neutrophilia is a key histologic feature of alcohol-associated steatohepatitis, and greater neutrophilic infiltration of the liver was observed in IL-6TS^+^ patients with available biopsies. Congruent with these findings, hepatic RNA expression of *FUT4*, a key neutrophil marker, was significantly higher in IL-6TS^+^ patients [median (IQR), 11.626 tpm (8.943–13.667) versus 2.610 tpm (1.910–5.480), *P* < 0.0001]. Using the IPA disease analysis tool, human liver RNA-seq data were analyzed for differences in pathophysiological functions between IL-6TS^+^ and IL-6TS^–^ patients (Table 5). Interestingly, the IL-6TS^+^ subset was enriched in pathways associated with leukocyte movement and functions, including cell migration and phagocytosis. Activation of the same leukocyte functions was identified from analysis of transcripts from hIL-6–stimulated HepG2 cells. Notably, genes encoding myeloid chemoattractants, including *SAA1, SAA2, LCN2*, and *LBP*, were enriched in both IL-6TS^+^ patients and in hIL-6–stimulated HepG2 cells. Together, these findings suggest that hepatocyte IL-6TS drives recruitment and activation of liver-infiltrating neutrophils in ALD.

**Table 5:** Ingenuity Pathway Analysis of Differentially Expressed Transcripts in the InTeam IL-6TS^+^ Subset and in Transcripts Induced after IL-6TS Stimulation of HepG2 Cells

Function	Differentially expressed transcripts in IL-6TS^+^ cohort (*n* = 3513 genes)		Transcripts induced by IL-6TS stimulation of HepG2 cultures (*n* = 390 genes)	
	Activation z score	*P*	Activation z score	*P*
Invasion of cells	3.863	1.73 × 10^−7^	5.344	3.10 × 10^−31^
Cell movement	2.821	1.02 × 10^−15^	7.12	1.64 × 10^−36^
Cellular infiltration by granulocytes	2.479	1.18 × 10^−05^		1.11 × 10^−13^
Cell movement of leukocytes	2.314	2.01 × 10^−08^	4.792	1.14 × 10^−13^
Migration of myeloid cells	2.285	9.27 × 10^−07^	5.091	1.11 × 10^−13^
Phagocytosis	2.284	4.35 × 10^−07^	4.314	4.90 × 10^−13^

### Ethanol Treatment Promotes STAT3 Activation and Neutrophilic Inflammation in Mouse Livers despite Reduced IL-6R Expression

**Figure 6:**
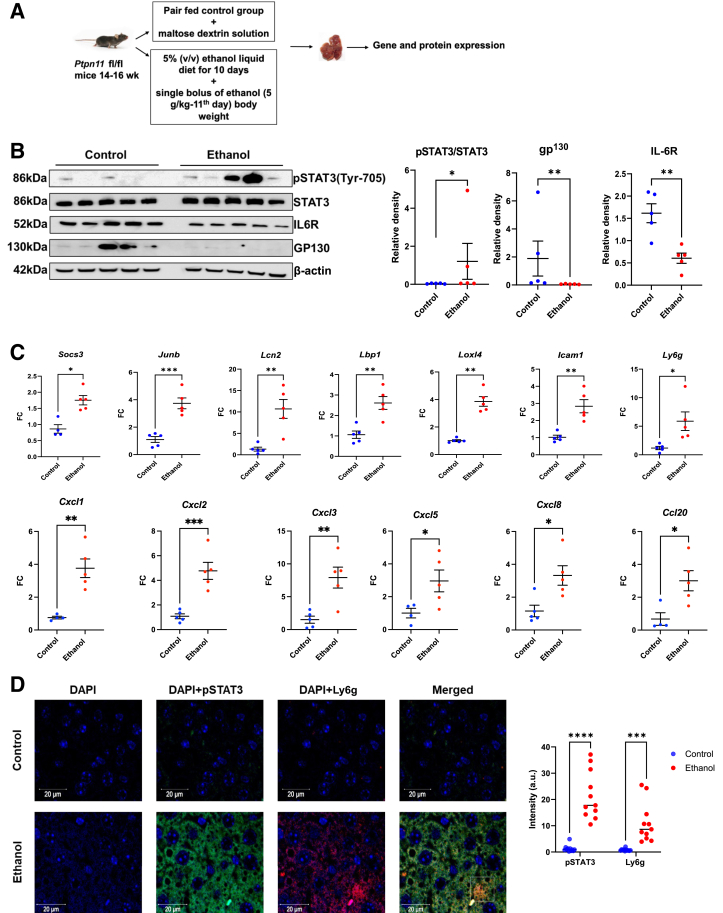
Ethanol treatment promotes STAT3 activation and neutrophilic inflammation in mouse livers despite reduced IL-6R expression. **A:** Female mice were fed a 5% (v/v) ethanol liquid diet (catalog number F1258SP; Bio-Serv, Flemington, NJ) *ad libitum* or pair-fed an isocaloric control liquid diet (catalog number F1259SP; Bio-Serv) for 10 days. On day 11, mice were administered a single bolus of ethanol (5 g/kg body weight) or isocaloric maltose dextrin solution by oral gavage. **B:** Immunoblot analysis of pSTAT3Y705, STAT3, IL-6R, gp^130^, and β-actin expression in pair-fed and ethanol-fed mice shown with densitometry. **C:** Quantitative real-time PCR gene expression of *Socs3, JunB, Lcn2, Lbp1, Icam1, Loxl4*, *Ly6g*, *Cxcl1*, *Cxcl2, Cxcl3*, *Cxcl5*, *Cxcl8*, and *Ccl20* in pair-fed and ethanol-fed mouse livers. **D:** Representative confocal images of DAPI (blue), pSTAT3 (green), Ly6G (red), and merged targets in pair-fed and ethanol-fed livers. Images are shown with quantification of immunofluorescence signal for pSTAT3 and Ly6G. *n* = 5 mice per group (**A**). ∗*P* < 0.05, ∗∗*P* < 0.01, ∗∗∗*P* < 0.001, and ∗∗∗∗*P* < 0.0001. Scale bars = 20 μm.

Hepatic IL-6 signaling activity was assessed in pair-fed mice and mice fed with 10 days of ethanol followed by 1 day of ethanol binge (Figure 6A). On protein immunoblot analysis of hepatic lysates, ethanol-treated mice exhibited markedly reduced protein expression of IL-6R and gp^130^ (Figure 6B). Despite this reduction, ethanol treatment enhanced STAT3 phosphorylation (Figure 6B) and expression of multiple STAT3-dependent genes, including regulators of STAT3 activation (*Socs3* and *JunB*), and key acute phase reactants associated with neutrophil activity (*Lcn2*, *Lbp1, Icam1,* and *Loxl4*); in addition, multiple neutrophil chemokines (*Cxcl1, Cxcl2, Cxcl3, Cxcl5, Cxcl8,* and *Ccl20*) were significantly increased in ethanol-treated mice (Figure 6C). Alcohol significantly increased the expression of the neutrophil marker Ly6G, as indicated by both qPCR and protein immunofluorescence (Figure 6, C and D). Importantly, increased Ly6G staining localized to regions adjacent to STAT3^+^ hepatocytes, indicating that Ly6G^+^ leukocytes were in contact with STAT3-activated hepatocytes (Figure 6D). Together, these findings suggest that IL-6TS may overcome ethanol-induced mIL-6R deficiency to enhance hepatocyte STAT3 activation and elaboration of neutrophilic activators, leading to neutrophilic infiltration in ASH.

## Discussion

The current study demonstrated that despite TGF-β1–mediated suppression of hepatic IL-6R expression in human AH, hepatocellular STAT3 activation increased with disease severity. The role of IL-6TS was then investigated as an alternative mode of hepatocellular STAT3 activation in AH. *In vitro* stimulation of IL-6TS in HepG2 cells generated a distinct gene signature enriched for acute phase reactants implicated in neutrophil chemotaxis and activation. This gene profile was subsequently used to identify a subset of patients with ALD in the InTeam cohort characterized by enhanced IL-6TS activity. Clinically, these patients exhibited a distinct clinical profile with more severe portal hypertension, liver dysfunction, peripheral leukocytosis, and intrahepatic neutrophilia. Furthermore, they exhibited up-regulated hepatic expression of genes associated with leukocyte migration and phagocytosis. In a murine model of ALD, ethanol increased murine hepatic STAT3 activation while suppressing IL-6R and gp^130^ expression, and multiple STAT3-dependent genes associated with neutrophil recruitment were significantly up-regulated by ethanol treatment. Finally, Ly6G^+^ leukocytes were in contact with STAT3-activated hepatocytes, suggesting that IL-6TS and hepatocellular STAT3 activation are key drivers of neutrophil recruitment and inflammation in AH.

TGF-β1 is a regulator of liver transcriptional reprogramming in AH.^23^ In the current study, TGF-β1 was the most potent inhibitor of IL-6R expression in human AH. Despite reduced hepatic IL-6R expression, however, STAT3 was the most activated hepatic transcription factor in severe AH. *In vitro*, only high-dose hIL-6 was able to overcome TGF-β1–mediated suppression of IL-6R expression to restore STAT3 activity. Comparative analysis of HepG2 gene expression profiles after *trans* or classical stimulation revealed that only the effect size of up-regulated genes was enhanced by *trans*-signaling, while there were no qualitative differences in actual genes expressed. Together, these findings suggest that TGF-β1 increases the STAT3 activation threshold by down-regulating mIL-6R, thus rendering hepatocytes reliant on *trans*-signaling for IL-6-dependent STAT3 activation in AH.

Excess neutrophilic infiltration into the liver exacerbates tissue inflammation in AH, as both neutrophil depletion and blockade of neutrophil migration via inhibition of intercellular adhesion molecule (ICAM)-1^35^ or E-selectin^12^ reduced liver injury in murine models. Hepatocytes have been increasingly recognized as key regulators of myeloid cell accumulation in the liver. In mouse models of alcohol-related liver injury, high levels of the neutrophil chemokine CXCL1 are released by TLR-2– and TLR-9–activated hepatocytes.^10^ Similarly, hepatocyte gp^130^ activation was sufficient to stimulate CXCL1 secretion, neutrophilic recruitment, and innate immune responses in murine models. Finally, IL-33 has also been proposed as a mediator of neutrophil accumulation, as damaged hepatocytes release IL-33 to regulate ST-2^+^ neutrophil chemotaxis in models of AH.^36^^,^^37^

In contrast, other studies have highlighted a role for STAT3 activation to orchestrate neutrophil infiltration in inflammatory liver diseases.^38^, ^39^, ^40^, ^41^, ^42^ Although many STAT3-dependent acute phase reactants are not direct neutrophil chemokines, these proteins can indirectly regulate neutrophil chemokine production and chemotaxis. In a murine study of liver metastasis, pancreatic cancer–derived IL-6 was found to activate hepatocyte STAT3, which in turn increased hepatocyte SAA1/SAA2 secretion and neutrophil accumulation with the metastatic niche,^38^ and SAAs direct neutrophil chemotaxis to sites of hepatic inflammation via *N*-formyl peptide receptor (FRP)-2 receptors.^39^^,^^40^ Furthermore, treatment of human neutrophils with SAA1/2 up-regulates neutrophil CXCL8 expression in an ERK1/2-dependent manner,^41^ and both whole SAA1 and naturally occurring cleaved SAA1 peptides synergistically enhance CXCL8-mediated neutrophil chemotaxis in murine models of sepsis.^32^^,^^42^ As a recent study demonstrated that severe AH is characterized by the accumulation of self-sustaining CXCL8^+^ neutrophils,^25^ SAA1/2 derived from hepatocyte IL-6TS may serve as a mechanistic link between STAT3 activation and neutrophil infiltration, and future studies are planned to assess neutrophil–hepatocyte interactions in AH.

Through *in vitro* models and in silico analyses of human RNA-seq data, it was observed that IL-6TS enhanced the expression of SAA1, SAA2, LCN2, and LPS-binding protein (LBP), and in murine models ethanol up-regulated the expression of *Lcn2* and *Lbp* in conjunction with L6yG^+^ leukocyte accumulation adjacent to STAT3-activated hepatocytes. Alcohol treatment stimulates hepatocyte STAT3 activation, LCN2 secretion, and neutrophil migration in mice, and this effect was ablated by pharmacologic inhibition or genetic ablation of STAT3.^43^ Neutrophils critically rely on LCN2 for chemotactic and phagocytic functions, as treatment of human neutrophils stimulates neutrophil chemotaxis *in vitro*.^44^, ^45^, ^46^ Furthermore, in rodent models of alcohol-related liver injury, hepatocyte-derived LBP increases intestinal permeability and facilitates LPS presentation to Kupffer cells via CD14.^47^^,^^48^ In other inflammatory diseases, including peritonitis and biliary obstruction, LBP is necessary for local production of the neutrophil chemokine macrophage migration inhibitory factor (MIF)-2 at sites of injury, and mice deficient in LBP exhibit reduced neutrophilic inflammation after tissue injury.^49^, ^50^, ^51^ Together, these findings suggest that hepatocyte IL-6TS coordinates neutrophil recruitment via multiple STAT3-dependent signaling pathways.

Although the current study primarily focused on the role of IL-6TS in hepatocytes in AH, it is important to recognize that both parenchymal and nonparenchymal hepatic cells express IL-6Rs. While the source of soluble IL-6R in AH remains unclear, it is plausible that cell type–specific production of soluble IL-6R may mediate interactions between different cell types to shape IL-6TS responses in AH. Future studies are planned to determine how cell type–specific sIL-6R generation affects crosstalk between subpopulations in AH.

Through complementary analyses of human liver specimens, *in vitro* hepatocyte models, and murine models of ALD, the current study identified IL-6TS as a key driver of hepatocyte STAT3 activation and neutrophilic inflammation in AH. These findings establish IL-6TS as a potential therapeutic target for the treatment of AH.

## Disclosure Statement

None declared.

## Supplemental Data

Supplemental Figure S1 Hierarchical clustering of generalized linear models of *IL6R* regulation from human RNA sequencing data demonstrated that clustering was dependent on liver disease severity. Disease severity cohorts included healthy controls (1), early alcohol-related steatohepatitis (ASH) (2), compensated alcohol-related cirrhosis (3), nonsevere alcohol-associated hepatitis (AH) (4), and severe AH (5). Results are depicted as a dendrogram with heatmap ranging from –3 (green), to 0 (black), to +3 (red).

Supplemental Figure S2 Epigenetic modifications are not involved in *IL6R* suppression in alcohol-associated hepatitis (AH). **A:** Genomic view of DNA methylation beta values in sequencing reads present in loci of *IL6R*; no significant difference in methylation patterns was observed between AH (*n* = 6) and healthy control (*n* = 5) liver tissue. **B:** Genomic view of histone marks (H3K27Ac, H3K4me1, and H3K4me3) in sequencing reads demonstrating no differences in chromatin structure at *IL6, IL6R, and IL6ST* gene loci between AH (*n* = 8) and control (*n* = 5) livers.

Supplemental Figure S3 Heatmap of differential gene expression in HepG2 cells. HepG2 cells were treated with TGF-β1 (5 ng/mL) overnight followed by stimulation with either 1% fetal bovine serum (control), IL-6 (20 ng/mL), or hIL-6 (20 ng/mL). Gene expression was quantified by RNA sequencing, normalized, and compared with nonstimulated controls. The most up-regulated genes by hIL-6 are shown as heatmap of log_2_ fold change.

Supplemental Figure S4 **A:** Hierarchical clustering of whole-liver transcriptomic data from an independent cohort of healthy controls and severe AH patients **B:** mRNA raw counts of *IL6*, *IL6R*, *IL6ST*, and *SOCS3* from IL-6 trans-signaling (TS)–high and IL-6TS–low livers. *n* = 7 (**A**, control); *n* = 13 (**A**, AH). ∗*P* < 0.05, ∗∗∗*P* < 0.001, and ∗∗∗∗*P* < 0.0001.
