# Supplementary figures and images for: IL-6 trans-Signaling Regulates Neutrophilic Inflammation in Alcohol-Associated Hepatitis
Source: Am J Pathol. 2025 Jun 23;196(1):223–40. doi: 10.1016/j.ajpath.2025.05.023 (PMC13168973; doi:10.1016/j.ajpath.2025.05.023)

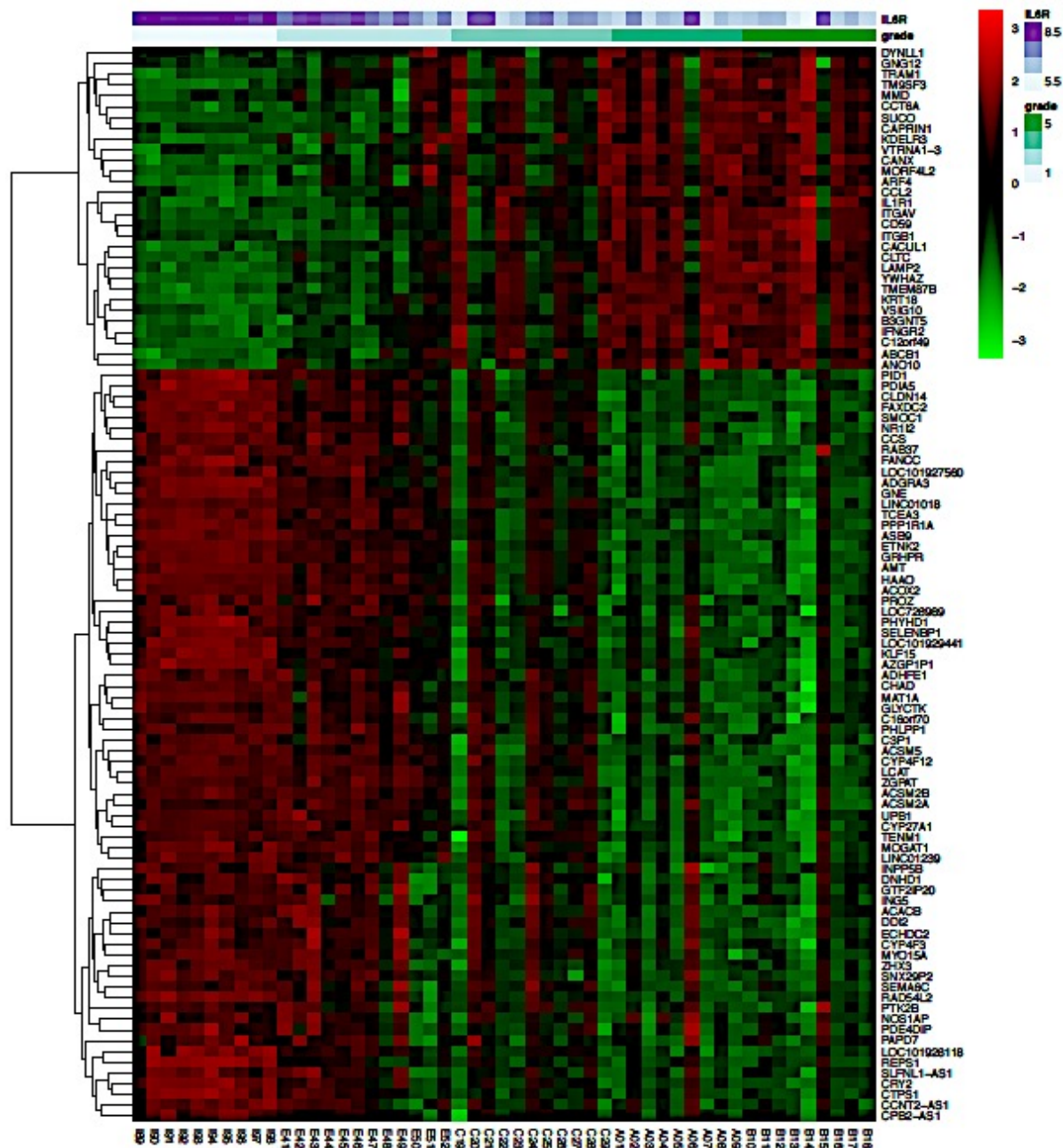

Supplement: Supplemental Figure S1 — Hierarchical clustering of generalized linear models of IL6R regulation from human RNA sequencing data demonstrated that clustering was dependent on liver disease severity. Disease severity cohorts included healthy controls (1), early alcohol-related steatohepatitis (ASH) (2), compensated alcohol-related cirrhosis (3), nonsevere alcohol-associated hepatitis (AH) (4), and severe AH (5). Results are depicted as a dendrogram with heatmap ranging from –3 (green), to 0 (black), to +3 (red). [file mmc1.pdf]

A

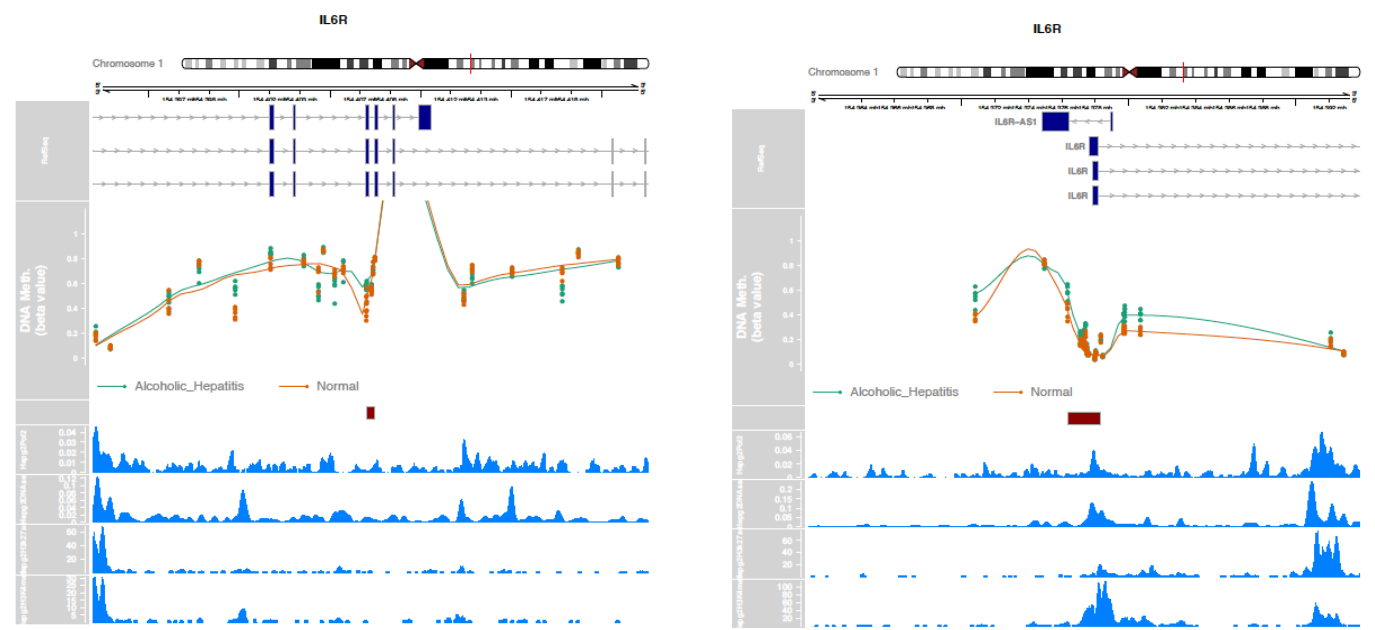

B

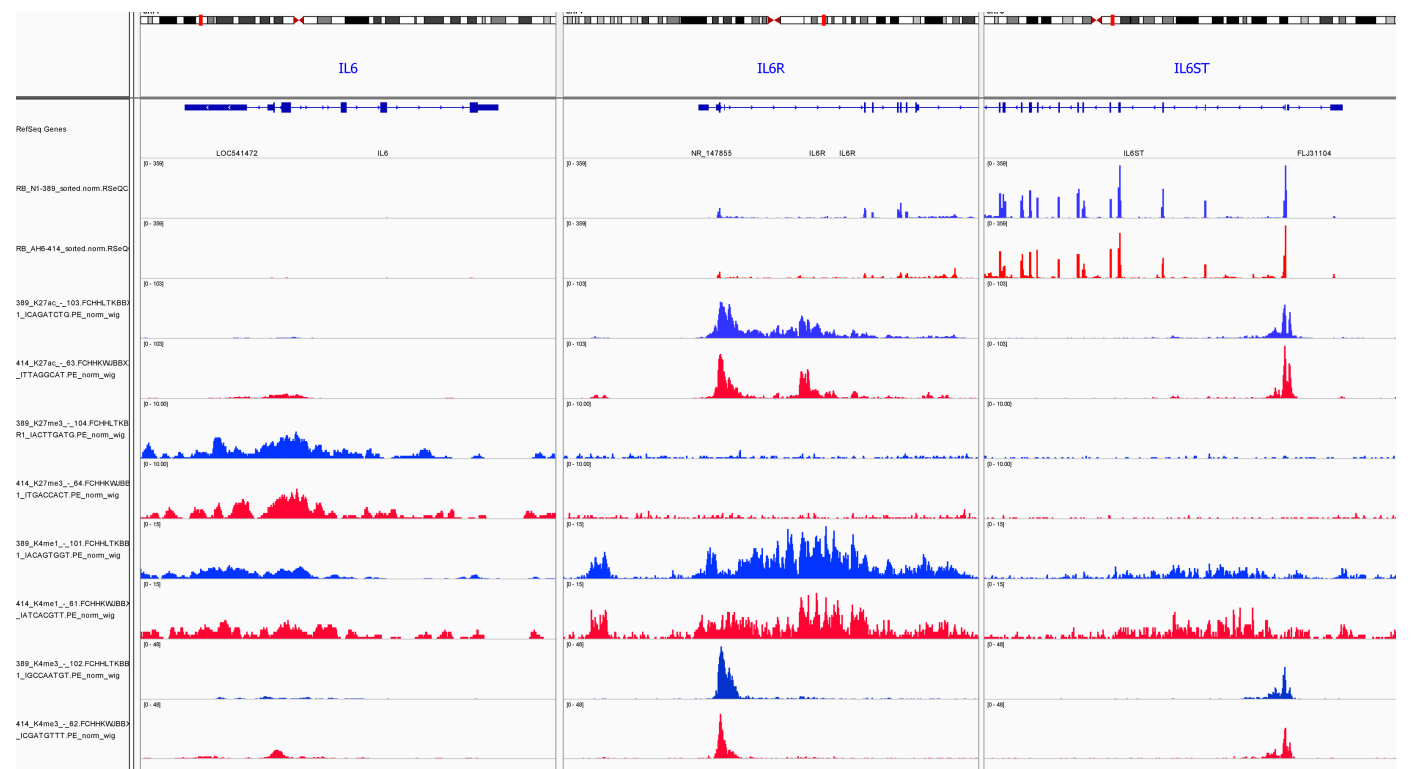

Supplement: Supplemental Figure S2 — Epigenetic modifications are not involved in IL6R suppression in alcohol-associated hepatitis (AH). A: Genomic view of DNA methylation beta values in sequencing reads present in loci of IL6R; no significant difference in methylation patterns was observed between AH (n = 6) and healthy control (n = 5) liver tissue. B: Genomic view of histone marks (H3K27Ac, H3K4me1, and H3K4me3) in sequencing reads demonstrating no differences in chromatin structure at IL6, IL6R, and IL6ST gene loci between AH (n = 8) and control (n = 5) livers. [file mmc2.pdf]

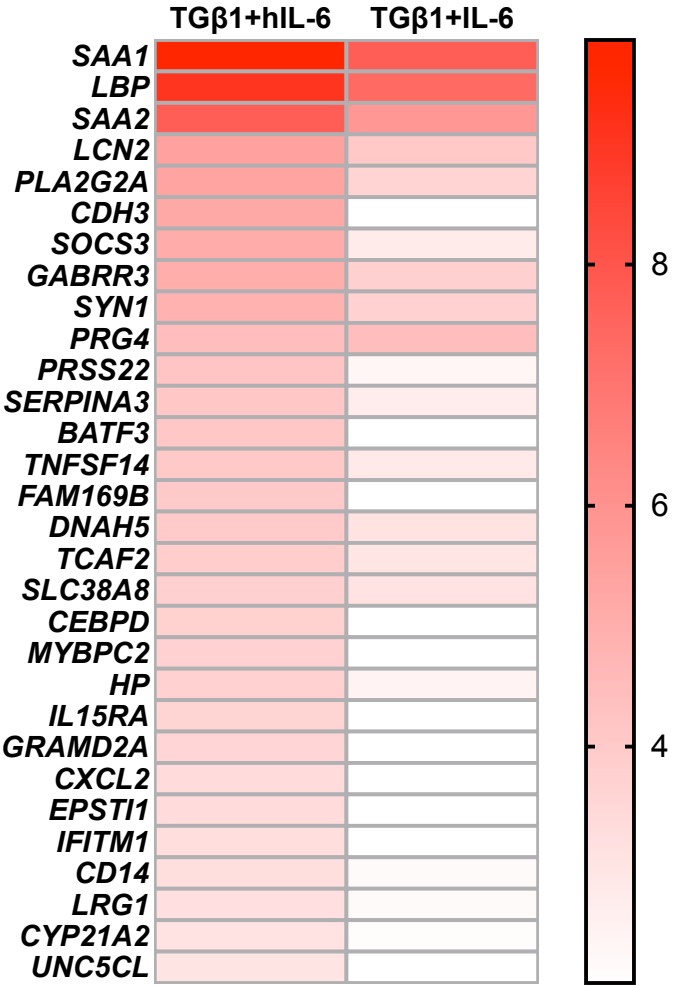

Supplement: Supplemental Figure S3 — Heatmap of differential gene expression in HepG2 cells. HepG2 cells were treated with TGF-β1 (5 ng/mL) overnight followed by stimulation with either 1% fetal bovine serum (control), IL-6 (20 ng/mL), or hIL-6 (20 ng/mL). Gene expression was quantified by RNA sequencing, normalized, and compared with nonstimulated controls. The most up-regulated genes by hIL-6 are shown as heatmap of log2 fold change. [file mmc3.pdf]

**A**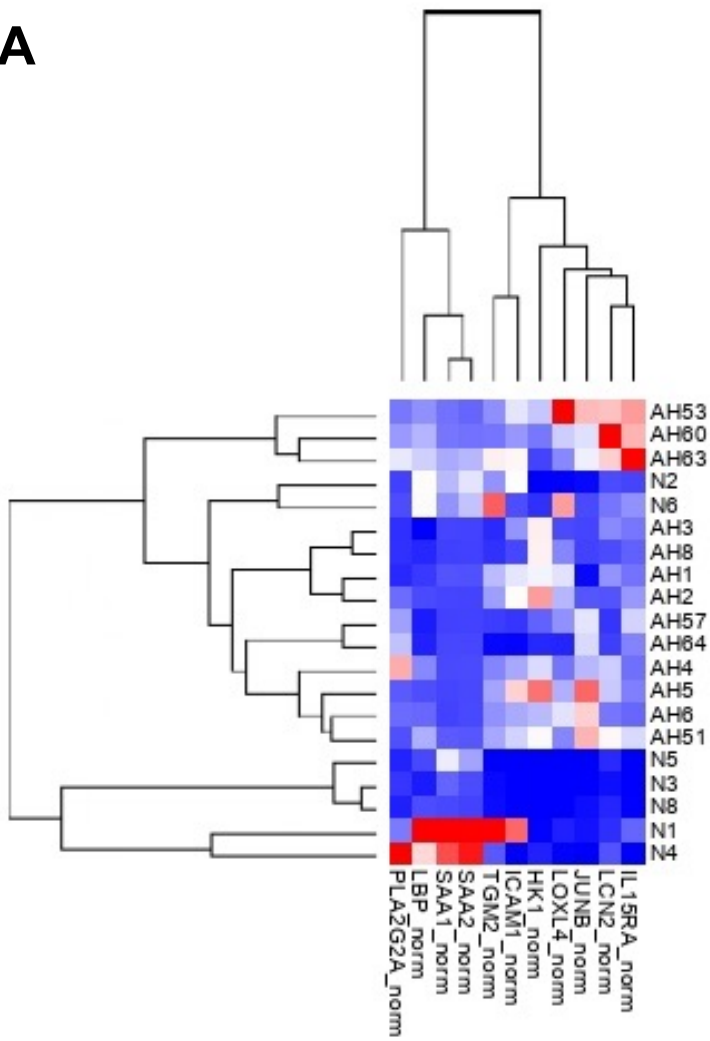**B**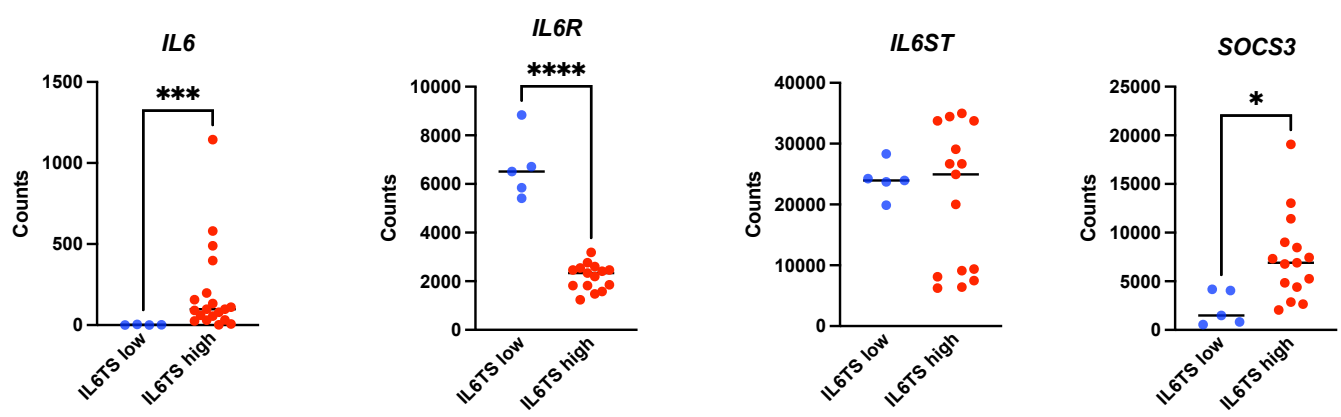

Supplement: Supplemental Figure S4 — A: Hierarchical clustering of whole-liver transcriptomic data from an independent cohort of healthy controls and severe AH patients B: mRNA raw counts of IL6, IL6R, IL6ST, and SOCS3 from IL-6 trans-signaling (TS)–high and IL-6TS–low livers. n = 7 (A, control); n = 13 (A, AH). ∗P < 0.05, ∗∗∗P < 0.001, and ∗∗∗∗P < 0.0001. [file mmc4.pdf]
